# Differential roles of regulatory T cells in acute respiratory infections

**DOI:** 10.1172/JCI170505

**Published:** 2023-07-17

**Authors:** Milica Jovisic, Nurbek Mambetsariev, Benjamin D. Singer, Luisa Morales-Nebreda

**Affiliations:** 1 Division of Pulmonary and Critical Care Medicine, Department of Medicine,; 2 Simpson Querrey Lung Institute for Translational Science,; 3 Division of Allergy and Immunology, Department of Medicine,; 4 Department of Biochemistry and Molecular Genetics, and; 5 Simpson Querrey Institute for Epigenetics, Northwestern University Feinberg School of Medicine, Chicago, Illinois, USA.

## Abstract

Acute respiratory infections trigger an inflammatory immune response with the goal of pathogen clearance; however, overexuberant inflammation causes tissue damage and impairs pulmonary function. CD4^+^FOXP3^+^ regulatory T cells (Tregs) interact with cells of both the innate and the adaptive immune system to limit acute pulmonary inflammation and promote its resolution. Tregs also provide tissue protection and coordinate lung tissue repair, facilitating a return to homeostatic pulmonary function. Here, we review Treg-mediated modulation of the host response to respiratory pathogens, focusing on mechanisms underlying how Tregs promote resolution of inflammation and repair of acute lung injury. We also discuss potential strategies to harness and optimize Tregs as a cellular therapy for patients with severe acute respiratory infection and discuss open questions in the field.

## Introduction

The COVID-19 pandemic, generations of influenza epidemics and pandemics, and the rise of multidrug-resistant bacterial pneumonia have increased awareness of the burden of acute respiratory infections (1, 2). Even before the COVID-19 pandemic, pneumonia due to a broad spectrum of viral and bacterial pathogens caused almost 80% of deaths from infection in the United States (3–5). The most common pathogens identified in patients with severe pneumonia are viruses and bacteria armed with a versatile set of tools to overwhelm and escape the host immune response (6). In addition to SARS-CoV-2, viruses frequently implicated in causing severe pneumonia include influenza viruses, respiratory syncytial virus (RSV), and rhinovirus (6, 7). Common pathogens recovered in bacterial pneumonia vary by the locale of acquisition, particularly community versus hospital settings, and include *Streptococcus pneumoniae* (pneumococcus), *Haemophilus influenzae*, *Staphylococcus aureus*, Enterobacterales, and *Pseudomonas aeruginosa* (1). Severe pneumonia from any etiology can result in the acute respiratory distress syndrome (ARDS), a heterogeneous clinical syndrome characterized by severe inflammation and injury to the alveolar epithelium and endothelium that is associated with mortality rates approaching 40% (8, 9). Across pathogens, a common thread in the pathophysiology of ARDS induced by severe pneumonia is a dysregulation of the immune response, which leads to aberrant inflammatory tissue damage (10–14). A delicate balance between an appropriate host response (to clear the infection) and processes that resolve inflammation and repair lung damage (to promote subsequent recovery) is tightly regulated by an ensemble of complex regulatory mechanisms of the innate and adaptive immune systems.

Regulatory T cells (Tregs) are a subset of CD4^+^ T cells that express the FOXP3 transcription factor and have diverse and context-specific functions in promoting immune homeostasis by suppressing over-exuberant immune system activation under steady-state and stressed conditions in the lung and other organs (15). In addition to maintaining self-tolerance, data mostly from mice demonstrate that Tregs exert important pro-resolution functions in the innate and adaptive immune response to pathogens, including those infecting the respiratory tract (16) (Table 1). Recently, multiple groups have described novel Treg functions in mice, distinct from their immunosuppressive roles, in promoting tissue protection and repair of tissue damage in the lung, muscle, skin, and vascular endothelium (15, 17–30). Less is known about the specific role of Tregs in resolution, tissue protection, and repair in human lung disease, but investigators have detected Tregs in the alveolar spaces of patients with pneumonia and ARDS (18, 31, 32). In this Review, we use “tissue-protective” to refer to processes that mitigate ongoing injury by imparting resilience to damage; “repair” refers to active processes that lead to regeneration of injured tissue. While these processes are often conflated in studies of immune-mediated tissue injury, they are separable events that require experimental interventions at different time points to accurately investigate. Translation of Treg-based therapeutics that limit immune-mediated tissue injury and promote tissue protection and repair following respiratory infection will require a detailed understanding of Treg identity and function (33). Here, our goal is to review distinct domains of Treg function in the host response to respiratory infection and subsequent lung injury and discuss open questions in the field that should be addressed as Treg-based therapeutics move into translation.

## Mechanisms of Treg development and function

Tregs exhibit robust expression of the high-affinity interleukin-2 (IL-2) receptor α subunit (CD25), and their generation and function are dependent on expression of the FOXP3 transcription factor (33). In vivo, Tregs can be classified into two main populations, thymus-derived Tregs (tTregs) and peripherally induced Tregs (pTregs), on the basis of their ontological origin. The tolerogenic role of tTregs is intricately linked to their development in the thymus; indeed, they arise from self-reactive thymocytes whose T cell receptor (TCR) recognizes self-antigen (34). Early events requiring the chromatin organizer SATB1 establish Treg-specific super-enhancer landscapes at the *Foxp3* conserved noncoding sequence (CNS) 0 and other loci that are necessary for Treg development before induction of *Foxp3* expression (35). Continuous TCR stimulation then activates transcription factors (e.g., nuclear factor-ĸB, nuclear factor of activated T cells, and forkhead box protein O) that directly bind to the promoter, CNS2, and CNS3 of the *Foxp3* gene to induce its expression (36). Signaling through the IL-2 receptor maintains the Treg-defining DNA methylation landscape through recruitment of ten-eleven translocation (TET) enzymes to CNS2 (37, 38). Ultimately, Tregs depend on this peculiar epigenetic landscape for their development and lineage stability (33, 39). In contrast, pTregs lacking the lineage-stabilizing tTreg-type epigenetic landscape arise from naive CD4^+^ T cells that mainly recognize non-self-antigens in peripheral tissues in response to transforming growth factor-β (TGF-β) and IL-2 (36).

Mutations in the *Foxp3* gene in mice result in the scurfy phenotype, which is characterized by a lymphoproliferative disorder and autoimmunity, including lymphocytic lung inflammation (40, 41). Similarly, human loss-of-function mutations in *FOXP3* cause the immune dysregulation, polyendocrinopathy, enteropathy, X-linked (IPEX) syndrome, which manifests with various autoimmune phenomena secondary to loss of self-tolerance across tissues, including the lung (42, 43). Accordingly, dysregulated Treg function underlies numerous autoimmune diseases, graft-versus-host disease, and allograft rejection syndromes in which the Treg immunosuppressive role negatively regulates pathogenic immune responses to self-antigen (44, 45). These immunosuppressive functions are mediated in part by secretion of immunomodulatory cytokines, including IL-10 and TGF-β (46–48), expression of inhibitory surface receptors such as CTLA-4 and PD-1 (49, 50), and adenosine generation in Tregs highly expressing CD39 and CD73 (51, 52). Tregs specific for tumor antigen inhibit the host antitumor immune response against malignant cells, rendering them a target for cancer immunotherapies (53, 54).

Mature Tregs can be classified based on distinctive phenotypic and functional attributes (55). Resting, or central, Tregs (cTregs) express CC chemokine receptor 7 (CCR7) and the adhesion receptor CD62L, which allow them to recirculate through secondary lymphoid organs (56). Activated, or effector, Tregs (eTregs) exhibit more phenotypic and functional adaptability, as they can upregulate specific transcription factors, chemokine receptors, and effector molecules, paralleling effector CD4^+^ T cell responses and enabling suppression of context-specific immunity (56). With the advent of next-generation sequencing technologies, investigators have shown how this conceptual framework incompletely accounts for the plasticity and functional heterogeneity of Tregs in different anatomical niches — allowing the identification of distinctive tissue-specific Tregs in the lung and other organs (57).

## Tregs suppress lung inflammation and promote resolution

**Figure 1:**
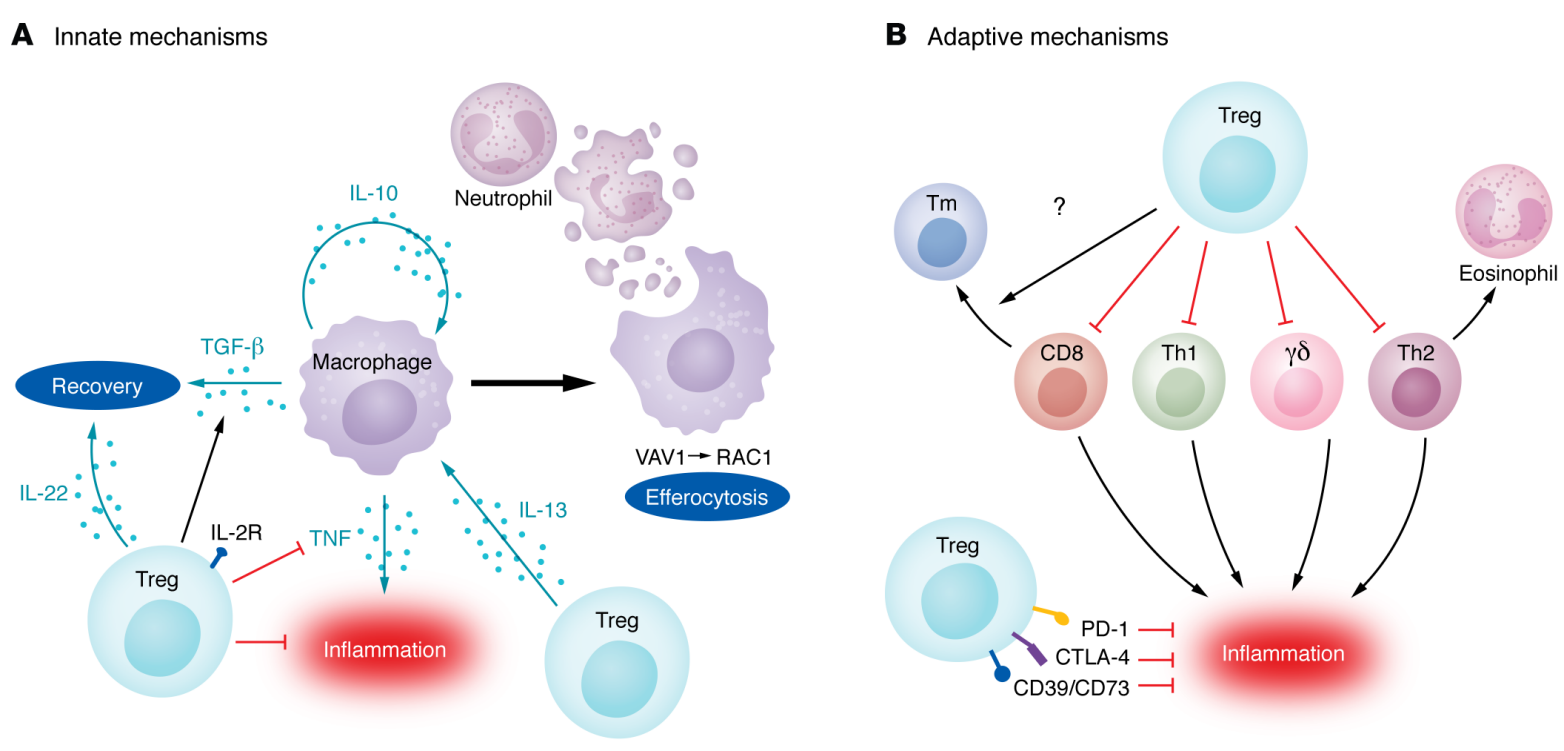
Mechanisms of Treg-mediated immunosuppression and resolution of lung inflammation. Tregs exert pleiotropic effects on innate (**A**) and adaptive (**B**) immune cells to dampen excessive alveolar inflammation and promote resolution following infection-induced lung inflammation and injury. (**A**) Tregs express the high-affinity IL-2 receptor and interact with other immune cell subsets, suppressing their activation and modulating their function. During resolution of lung inflammation, Tregs generate IL-13, signaling alveolar macrophages to secrete IL-10. Autocrine IL-10 then promotes macrophage efferocytosis of apoptotic neutrophils through activation of the VAV1/RAC1 signaling pathway and subsequent cytoskeleton remodeling to prevent excessive tissue damage. Tregs also promote a pro-resolution phenotype in macrophages by inhibiting TNF-α generation and inducing TGF-β secretion. IL-22 induction, possibly through a CD4^+^ Th cell intermediate, contributes to recovery and may be Treg dependent. (**B**) Tregs also dampen primary T cell responses to respiratory infections that include CD4^+^ Th1 cells, Th2 cells (and subsequent recruitment of eosinophils), CD8^+^ T cells, and γδ T cells, inhibiting excessive inflammation in response to pathogens. In contrast, Tregs may have a role in the generation of CD8^+^ memory cells, but their effect on individual compartments of the memory pool is unknown. Tregs also express surface molecules, such as CTLA-4, PD-1, CD39, and CD73, that inhibit inflammation, but the role of these molecules in responses to lung pathogens is unclear.

As noted above, Tregs function to suppress excessive immune system activation and maintain immune homeostasis (15). Following acute lung injury due to a respiratory pathogen, an initial innate immune response leads to robust neutrophil recruitment to the lung. Neutrophils serve multiple roles in pathogen clearance, including ingestion and killing of opsonized pathogens, generation of microbicidal reactive oxygen species and peptides, secretion of tissue-remodeling enzymes, and the elaboration of neutrophil extracellular traps (58). While these functions serve to promote pathogen clearance, particularly in bacterial and fungal pneumonia, unrestrained and persistent tissue neutrophilia results in excessive lung tissue damage, respiratory failure, and poor clinical outcomes (58, 59). Hence, neutrophil efferocytosis — the phagocytic clearance of dead and dying neutrophils — represents a key event in the resolution of lung inflammation. Tregs interact with resident and recruited alveolar macrophages to promote neutrophil efferocytosis and hasten recovery (18, 60) (Figure 1). Mechanistically, experiments in mice determined that Tregs generate IL-13 to stimulate lung macrophage production of IL-10, which signals via the VAV1/RAC1 pathway in an autocrine manner to promote efferocytosis during resolution of lung inflammation (60). Activation of other Treg pathways that promote neutrophil efferocytosis following bacterial clearance could limit lung inflammation and expedite host recovery. Bronchoalveolar lavage–based (BAL-based) strategies to define the timing of pathogen clearance in patients with severe pneumonia may help determine the time window when pro-efferocytosis pathways can be safely induced without sacrificing pathogen-clearing immune system functions (61).

In addition to promoting macrophage efferocytosis of apoptotic cells, Treg-macrophage crosstalk modulates other functions of lung macrophages during the initiation and resolution of pulmonary inflammation and injury (reviewed in ref. 62). Coculture experiments using mouse Tregs and lipopolysaccharide-stimulated (LPS-stimulated) alveolar macrophages revealed that Tregs function to decrease macrophage TNF-α generation in a contact-dependent manner (18). Similar experiments using thioglycolate-induced peritoneal macrophages demonstrated that Tregs also promote macrophage generation of the pro-resolving yet potentially profibrotic cytokine TGF-β (18). In the mouse intratracheal LPS model of acute lung injury, Treg-mediated recovery is dependent on TGF-β (18). In contrast, genetic experiments revealed that Treg generation of another antiinflammatory cytokine, IL-10, appears to be dispensable for resolution in the LPS model (18), although, as noted above, Tregs may signal to alveolar macrophages to promote macrophage secretion of pro-resolving IL-10 (63). IL-22 is a cytokine of the IL-10 family that signals to fibroblasts and epithelial cells to promote pulmonary antiviral responses (64), and some severe lung infections have been linked to parallel defects in IL-22 and Treg function (65). Interestingly, experimental data in mice suggest that FOXP3 suppresses IL-22 production in Tregs but that Tregs promote T helper (Th) cell production of IL-22 (66). Finally, lipid mediators in the leukotriene B_4_/BLT1 pathway recruit Tregs to the alveolar space and promote resolution of lung inflammation (67), suggesting that modulators of leukotriene function could promote Treg recruitment and function following lung infection. A detailed assessment of Treg-macrophage interactions and Treg-generated cytokine profiles in humans with lung infection will promote our understanding of how these dynamics modulate the immune response to drive outcomes in patients. For example, we performed serial sampling via BAL in mechanically ventilated patients with severe pneumonia to reveal complex T cell–macrophage interactions that were mediated by specific cytokine and chemokine signaling loops in COVID-19 (32, 68) and targetable in patients using a small-molecule inhibitor of calcium release–activated calcium channels (69).

The role of Tregs in modulating lung inflammation mediated by the adaptive immune system is context dependent. Antigen-specific Tregs generated following infection attenuate the immune response against the pathogen, limiting damage from inflammation but also potentially contributing to chronic infections in the lung and other organs (70–73). Following influenza infection, viral antigen–specific Tregs are generated and limit bystander CD4^+^ T cell responses (74). Specific requirements for viral antigens in the Treg response to virus-induced lung injury remain unclear. The timing of induction of Treg functions also affects the phenotype of the adaptive immune response. While Treg depletion early during human metapneumovirus infection inhibits priming of CD8^+^ T cell responses, late depletion is dispensable (75). In a mouse model of pneumococcal pneumonia, a subset of TNFR2-expressing Tregs dampens bacterial dissemination by inhibiting proinflammatory IL-17A secretion by γδ T cells (76). In RSV infection, Tregs inhibit virus-specific CD4^+^ and CD8^+^ T cell responses to prevent excessive inflammation and immunopathology, including eosinophil-mediated inflammation (77, 78). Treg suppressive function is further highlighted in influenza vaccination, during which vaccine-generated, antigen-specific Tregs attenuate the adaptive response to immunization, decreasing the protective effect of the inoculation (79). Interestingly, Tregs promote optimal CD8^+^ memory T cell responses following influenza infection that are protective following a secondary challenge (80). The effect of Tregs on the tissue-resident memory T cell (Trm) compartment in the lungs is not known, although type 1 regulatory T cells — an antigen-tolerizing, unconventional subset of Tregs — are required for maintenance of the Trm pool in the gastrointestinal tract (81).

Ultimately, promoting Treg functions that allow pathogen clearance by other immune system cells while limiting harmful tissue inflammation will augment host recovery from infection and lung injury. Although the optimal balance and timing of Treg immunosuppressive functions that limit and resolve inflammation remain undefined, it is encouraging that data from human Treg cellular therapy trials in graft-versus-host disease have not shown a signal indicating an increased risk of severe infection (82, 83). Going forward, optimization of beneficial immunosuppressive functions of Tregs, perhaps via ex vivo strategies discussed below, will facilitate their deployment in the clinical setting.

## Tissue protection and repair following infection-induced lung injury

**Figure 2:**
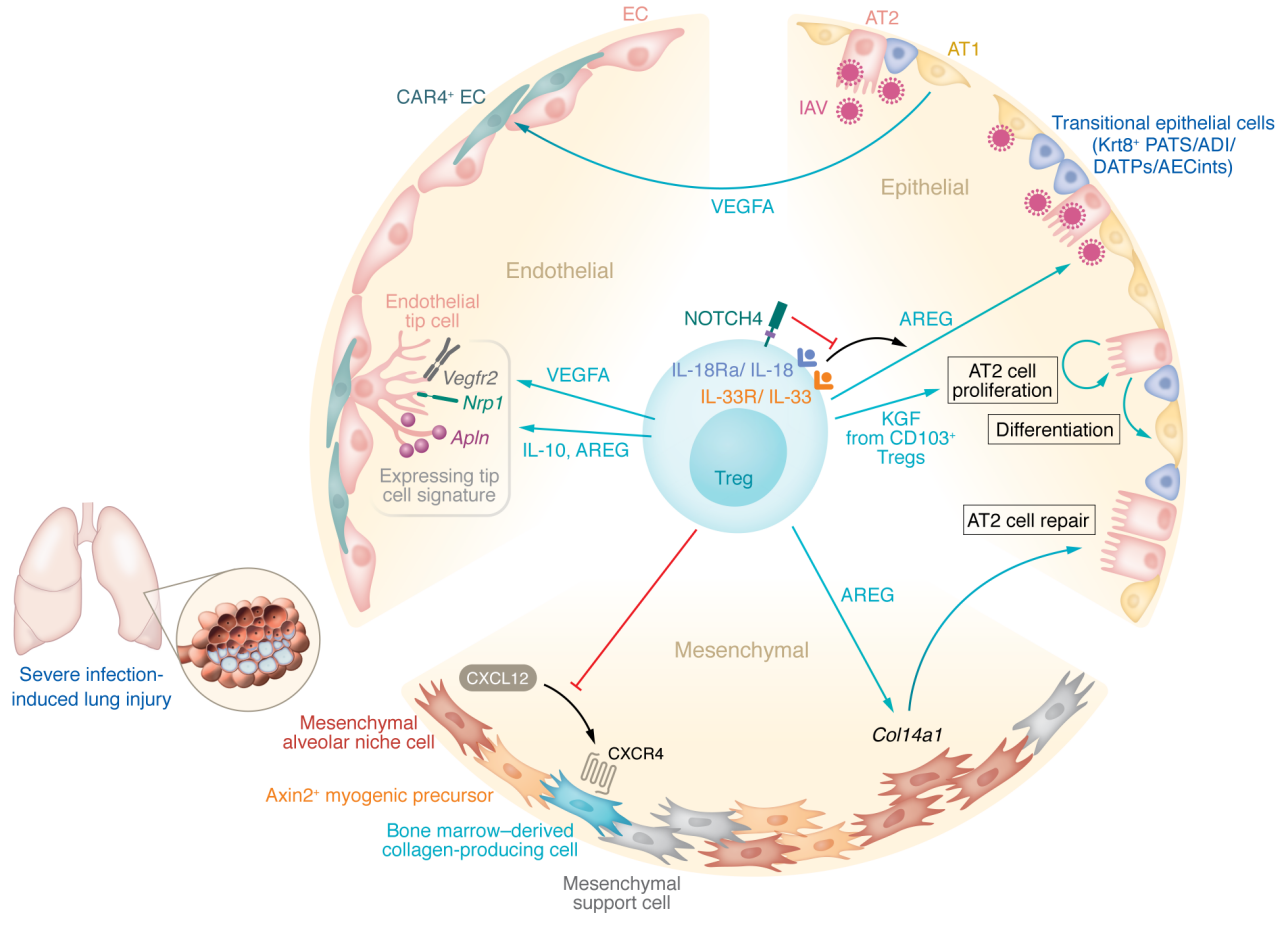
Mechanisms of Treg-mediated lung parenchymal tissue protection and repair. Tregs exert distinct functions to provide tissue protection and promote repair of the epithelial, endothelial, and mesenchymal compartments following infection-induced lung injury. Following lung injury, for example from influenza A virus (IAV) infection, signals from activated immune cells and damaged epithelial cells (e.g., IL-18 and IL-33) bind to their receptors on Tregs and drive Treg production of pro-epithelial growth factors (e.g., AREG and KGF) that signal to epithelial and *Col14a1^+^* mesenchymal cells to promote epithelial regeneration. NOTCH4 regulates this axis in Tregs. The role of Tregs in modulating the population of KRT8^+^ transitional epithelial cells — also known as pre-alveolar type 1 transitional cell state (PATS), alveolar differentiation intermediate (ADI), damage-associated transient progenitors (DATPs), and intermediate alveolar epithelial cells (AECints) — remains unclear. Tregs also generate pro-endothelial growth factors such as VEGF that promote the regeneration of alveolar capillary endothelial cells (ECs), including those expressing carbonic anhydrase 4 (CAR4) and the endothelial tip cell markers VEGFR2, NRP1, and APLN. Tregs also decrease fibroproliferation by decreasing signaling along the CXCL12/CXCR4 axis to limit collagen deposition by bone marrow–derived collagen-producing cells. AT1 cell, alveolar epithelial type 1 cell; AT2 cell, alveolar epithelial type 2 cell.

The respiratory tract is remarkably quiescent despite being continuously exposed to inhaled pathogens and air particles that challenge the homeostatic resilience mechanisms that maintain normal lung architecture and physiology (84, 85). Hence, during an over-exuberant stimulus (e.g., infection-induced ARDS) that disrupts tissue integrity and normal function, the lung must tightly balance cellular mechanisms that clear pathogens, preserve healthy tissue, and activate facultative cellular progenitors that regenerate damaged tissue (86). To exert these myriad functions, the lung is composed of distinctive cell populations within interdependent anatomical regions corresponding to epithelial, endothelial, and mesenchymal compartments (87) (Figure 2). In humans and mice, Tregs accumulate and expand in the alveolar space in response to injury (18–23, 25–27, 88). Aside from their capacity to resolve inflammation, Tregs establish intercellular circuitries that orchestrate compartment-specific tissue protection and repair of the respiratory system.

In conjunction with alveolar epithelial type 1 (AT1) cells, alveolar epithelial type 2 (AT2) cells form a tight barrier that lines the alveolar epithelium. AT2 cells secrete surfactant to facilitate lung expansion and prevent atelectasis, absorb excess alveolar fluid through vectorial ion transport, and, in response to injury, exhibit stem/progenitor cell–like properties, including self-renewal capacity and transdifferentiation into AT1 cells to reconstitute the epithelial side of the alveolar gas-exchange barrier (85). In experimental inflammatory and non-inflammatory mouse models of lung injury and regenerative alveologenesis, Mock and colleagues demonstrated that Tregs expressing CD103 (α_E_ integrin) promote AT2 cell proliferation (20) and that Treg depletion alters AT2 cell transcriptional profiles (24). Following on this observation, they went on to demonstrate that the epithelial growth factor keratinocyte growth factor (KGF) mediates Treg-specific enhancement of AT2 cell proliferation in mice (21). Many of these studies used diphtheria toxin to deplete Tregs in mice whose Tregs express the human diphtheria toxin receptor (89). In these mice, prolonged absence of Tregs leads to spontaneous inflammation and tissue injury, possibly confounding experiments designed to test specific tissue-protective and reparative Treg functions. Although the mouse epithelial growth factor receptor (EGFR) has low affinity for diphtheria toxin (90), subtle blockade of the mouse EGFR by diphtheria toxin could contribute to injury and dysregulated repair in these systems. In patients with ARDS, intravenous administration of KGF did not promote recovery and may have been harmful in a phase II randomized controlled trial (91). It remains possible, however, that lung Tregs programmed to overexpress KGF could promote epithelial repair via local and contextual cell-cell interactions that were not achieved by an intravenous infusion.

Production of growth factors such as the EGFR ligand family molecule amphiregulin (AREG) has emerged as a mechanism of Treg-mediated tolerance, tissue protection, and possibly active repair, in multiple tissue types, including the lung (23, 28, 92–97). Specifically, in a mouse model of intranasal influenza-induced lung injury, Arpaia and colleagues found that IL-18 and IL-33 signaling drives Treg production of AREG to maintain lung gas exchange function and barrier architecture, suggesting a tissue-protective and perhaps reparative role for Treg-derived AREG (23). The signaling and transcriptional pathways by which Tregs exert reparative functions in the lung are now beginning to be uncovered. NOTCH4 regulates IL-18–induced generation of AREG by Tregs in mice with experimental lung injury, and NOTCH4 expression is inversely associated with serum AREG and disease severity in patients with COVID-19 (97). Recently, Kaiser and colleagues identified a *Col14a1*-expressing mesenchymal cell population that is particularly sensitive to Treg-derived AREG, colocalizes with infiltrating Tregs during influenza-induced lung injury in mice, and supports alveolar epithelial organoid growth (96). In that study, genetic ablation of EGFR activation on stromal cells resulted in worsening oxygenation in mice following influenza infection, suggesting that Treg-derived AREG exerts — to some extent — its tissue-protective effect through mesenchymal cells. Importantly, the mesenchymal lineage of the lung is composed of distinct subpopulations with unique spatial distribution and regulatory functions. Whereas some mesenchymal cell subsets support alveolar epithelial growth, others (e.g., AXIN2^+^ myogenic precursors) drive dysplastic injury responses through myofibroblast activation and collagen deposition (98). During the fibroproliferative phase of experimental acute lung injury in the LPS mouse model, Tregs reduce recruitment of bone marrow–derived collagen-producing cells by decreasing signaling via the CXCL12/CXCR4 axis to mitigate postinjury lung fibrosis (99). In that study, the CXCR4 antagonist AMD3100 decreased fibroproliferation independent of CXCL12 levels in the lung, credentialing CXCR4 as a target in fibroproliferative lung injury.

Restoration of gas exchange function after pneumonia-induced lung injury also requires repair of the alveolar endothelium. As discussed above, the mechanisms of Treg-mediated alveolar epithelial repair following injury have been the focus of numerous experiments, but these studies have largely ignored the necessity of simultaneous endothelial repair to restore lung homeostasis. It is increasingly recognized that repair of the alveolar capillaries requires coordinated signals between the endothelium and epithelium (100). Indeed, during developmental and postviral lung injury conditions, different groups of investigators identified a distinctive lung endothelial cell (EC) population characterized by carbonic anhydrase 4 (CAR4) expression and dependence on epithelial cell–derived VEGFA (101, 102). Notably, CAR4^+^ ECs are anatomically juxtaposed to AT1 cells, proliferate in areas of severe postviral inflammation, and exhibit a transcriptional signature enriched for endothelial tip cell genes (e.g., *Vegfr2*, *Nrp1*, and *Apln*), underscoring a potential key role for this EC population in the coordination of intercellular signaling networks during regenerative alveolar remodeling. The role of Tregs in promoting vessel sprouting (angiogenesis) has been explored mostly in the context of tumor immunity and tissue-specific ischemic injury (103). Facciabene and colleagues reported that hypoxic intraperitoneal tumors upregulate CCL28 to recruit CCR10^+^ Tregs (104). Once in the tumor microenvironment, these Tregs promote EC expansion through increased VEGFA expression. In a mouse model of type 2 diabetes mellitus–induced peripheral artery disease, Tregs facilitate de novo formation of blood vessels through release of AREG and IL-10 in an apelin-dependent manner (105). Finally, in the mouse lung, D’Alessio and colleagues demonstrated that Tregs were necessary to mediate lung angiogenesis, but the mechanisms by which Tregs exert this reparative function remain unclear (106).

Age is the predominant risk factor for lung diseases, including pneumonia, and is associated with progressive homeostenosis — a lack of pro-homeostatic physiological reserves. Before the COVID-19 pandemic, influenza viruses caused up to 650,000 respiratory deaths per year worldwide, mostly in people over age 65 (107). Recently, the COVID-19 pandemic has highlighted the dramatic association between age and the severity of viral pneumonia–induced ARDS (108). Adaptive T cell immune competence wanes throughout the lifespan, as does the naive T cell repertoire and T cell–specific protective and reparative functions (109, 110). Our group demonstrated in a mouse model of heterochronic adoptive Treg transfer that old Tregs exhibit a cell-autonomous impairment in their ability to promote resolution of lung inflammation and parenchymal repair following viral pneumonia (25). In that study, lung Tregs in young adult mice upregulated AREG, among other reparative molecules, to a greater extent than lung Tregs from old mice following influenza infection. Age-related alterations in DNA methylation patterning are a core hallmark of aging (108), and in our study, genome-wide DNA methylation profiling revealed that age-related epigenetic alterations explained the loss of reparative transcriptional programs following influenza infection. Beyond the lung, a growing body of literature suggests that age-related Treg dysfunction affects other tissues and organs, including adipose tissue, skeletal muscle, and the nervous system (29, 111, 112). In addition to age, sex and gender are important biological and demographic variables, respectively, that are associated with differential risks from severe pneumonia, including due to SARS-CoV-2, with male sex and gender consistently demonstrating increased susceptibility (113–115). Experimental data in the mouse model of pneumococcal pneumonia demonstrated that estrogen signaling promotes Treg-macrophage crosstalk to restrain macrophage proinflammatory responses (116). Going forward, a more detailed understanding of the cellular and molecular mechanisms underpinning age- and sex-related alterations in Treg phenotype and function will better inform the development of Treg-targeted or Treg-based immunotherapies that promote recovery from severe respiratory infections across populations.

## Tregs as clinical immunotherapy

While few studies have leveraged Tregs as a cellular therapy for human lung diseases, proof-of-concept studies demonstrated the safety of Treg infusions in suppressing inflammation associated with autoreactive disorders, including type 1 diabetes mellitus, graft-versus-host disease, and organ allotransplantation (83, 117–122). Because of their pleiotropic beneficial effects on infection-related acute lung injury, approaches to administer Tregs as a pro-recovery therapy have gained momentum during the COVID-19 pandemic, with early-phase studies suggesting safety of allogeneic Treg infusions for patients with severe SARS-CoV-2 pneumonia (27, 123). Most human trials have used polyclonal autologous or allogeneic Tregs isolated from peripheral whole blood or umbilical cord blood via immunomagnetic systems or flow cytometry cell sorting (33, 124). These protocols usually involve ex vivo expansion of Tregs to generate sufficient numbers for infusion (up to 5 × 10^9^ cells per dose, which typically take 2–5 weeks to generate in culture). The addition of compounds such as rapamycin that suppress conventional T cell growth while promoting growth of Tregs may enhance Treg purity during ex vivo expansion (125, 126). An important barrier to using ex vivo–expanded autologous Tregs for the treatment of severe acute lung injury is that critically ill patients are often not capable of providing sufficient blood or cells, and the acuity of pneumonia-induced ARDS limits the time window to wait for ex vivo Treg expansion. Hence, optimizing allogeneic Treg products and developing strategies that maximize the on-target efficacy and safety of limited numbers of infused Tregs represents an important goal for the field. Moreover, tailoring Treg cellular therapy to exploit specific immunosuppressive versus tissue-protective and reparative functions could benefit clinical contexts characterized by persistent inflammation or injury, respectively.

**Figure 3:**
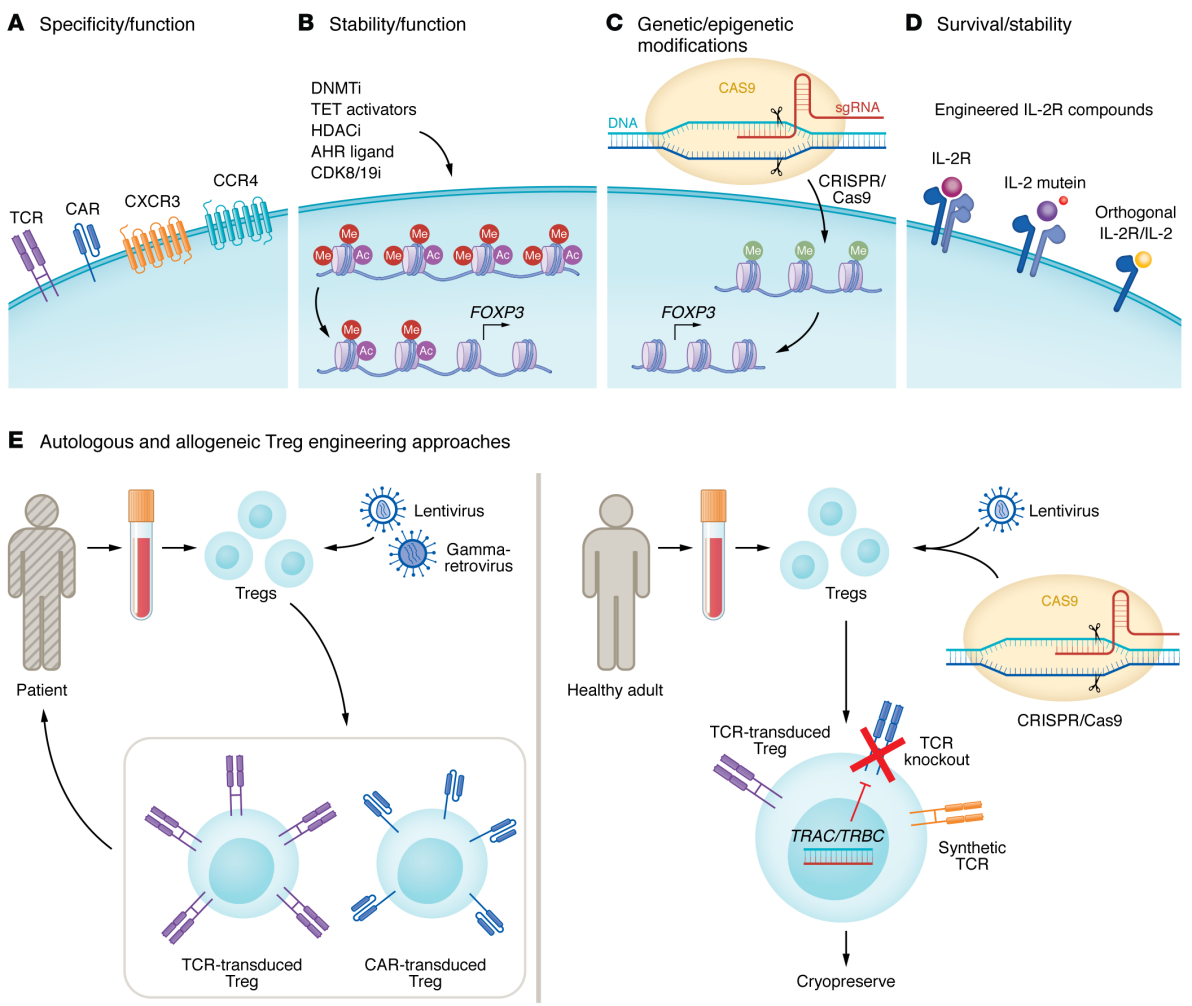
Potential strategies to promote the efficacy and safety of Treg cellular therapies for patients with infection-induced lung injury. (**A**) Tregs can be genetically engineered to express a synthetic receptor — artificial T cell receptor (TCR), chimeric antigen receptor (CAR), C-X-C motif chemokine receptor 3 (CXCR3), or CC chemokine receptor 4 (CCR4) — that recognizes an antigenic target of interest or enhances trafficking to the inflamed lung. (**B**) Pharmacological modifiers, including DNA methyltransferase inhibitors (DNMTi), ten-eleven translocation (TET) activators, histone deacetylase inhibitors (HDACi), aryl hydrocarbon receptor (AHR) ligands, and inhibitors of cyclin-dependent kinases 8 and 19 (CDK8/19i), could enhance the stability and function of infused Tregs. (**C**) Genetic/epigenetic modification using CRISPR-based technologies could also promote Treg-specific expression programs and improve the safety and efficacy of Treg-based therapies. (**D**) Engineered IL-2 proteins and receptors can be used to improve the specificity of IL-2 therapy, thus extending Treg survival. IL-2 muteins harbor targeted mutations that limit binding to the dimeric IL-2 receptor, while preserving binding to the high-affinity trimeric IL-2 receptor in Tregs. Engineered orthogonal IL-2 protein only binds engineering-generated orthogonal IL-2R Tregs, thus allowing selective proliferation and survival of these cells. (**E**) Autologous Tregs are virally transduced to express a synthetic CAR or engineered TCR that could promote antigen specificity and trafficking after therapeutic infusion. Allogeneic Tregs are isolated from healthy people; to limit alloreactivity, genome-editing technologies could be used to remove endogenous TCRs and replace them with a synthetic TCR that confers increased specificity and potency.

Advances in the fields of synthetic immunology, cytokine biology, immunopharmacology, and cutting-edge genome and epigenome editing technologies have broadened the capacity to engineer Tregs with enhanced trafficking, specificity, context-specific function, survival, and stability (Figure 3). CCR4-expressing CD103^+^ Tregs are important for lung-specific recruitment of Tregs, as CCR4-deficient Tregs have limited lung trafficking capabilities, resulting in pneumonitis (127). Expression of the T helper type 1–specifying (Th1-specifying) transcription factor T-BET in Tregs drives expression of the chemokine receptor CXCR3 to promote trafficking to sites of Th1-skewed inflammation (128). Engineered Tregs with specific homing programs could target their function to sites of Th1 inflammation such as the acutely infected and inflamed lung. Chimeric antigen receptor (CAR) Tregs have been developed as a therapeutic strategy in autoimmune disease (129–131). After collection of Tregs from healthy donors or ex vivo generation/expansion, Tregs can be engineered to express synthetic receptors such as CARs or specific TCRs to confer specificity and potentially enhance reparative function in the lung. As they are engineered for antigen specificity, CAR Tregs or those with modified TCRs have the potential to decrease the number of cells required for therapeutic efficacy. Additionally, CRISPR-based strategies to delete or modify the Treg TCR could limit unintended autoreactivity of transferred Tregs. Further studies are needed to test the applicability of engineered Tregs in the setting of respiratory infections where Treg function is variably dependent on antigen recognition (see “Open questions” section below).

Some evidence suggests that FOXP3^+^ cells exhibit plasticity in inflamed and damaged microenvironments, leading to loss of their suppressive functions and gain of proinflammatory effector functions (132). Hence, a period of ex vivo expansion provides an opportunity to pharmacologically modify Tregs in ways that support their functional stability with the potential to augment desirable antiinflammatory and pro-repair functions (33). Epigenetic modifiers that target histone deacetylases, DNA methyltransferases, and TET demethylases may stabilize Tregs before therapeutic infusion (19, 133–140). Experimental data suggest that aryl-hydrocarbon receptor ligands (141, 142) and cyclin-dependent kinase 8 (CDK8) and CDK19 inhibitors (143–145) also promote Treg identity and function. Applying these modifiers in a goal-driven fashion could optimize Treg lineage stability and function prior to infusion, limiting off-target effects and maximizing intended benefits.

Postinfusion strategies that enhance Treg stability and function may also promote on-target effects of therapeutically transferred Tregs. Tregs depend on signaling through the high-affinity IL-2 receptor α subunit (CD25) for their development (146, 147), and approaches to augment Treg function by administering exogenous IL-2 have been successful in inducing tolerance, for example, in a trial of patients with graft-versus-host disease (148). While IL-2 administration can have toxic effects — including venous thromboembolism, capillary leak, and activation of effector T cell responses due to the expression of variable-affinity IL-2 receptors on endothelial, T, and NK cells (149) — low-dose IL-2 administration was well tolerated in that trial. To mitigate the potential toxicities associated with higher-dose exogenous IL-2 administration, investigators have applied gene editing technologies to enhance the Treg specificity of IL-2 therapy. Transduction with an orthogonal IL-2 receptor that selectively responds to orthogonal IL-2 rather than native IL-2 could specifically expand and maintain the transferred Treg population without binding ECs or expanding effector T and NK cells (150, 151). Other engineered IL-2–based compounds, including IL-2 muteins, IL-2/anti–IL-2 immune complexes, PEGylated IL-2, and IL-2–CD25 fusion proteins, may also boost in vivo Treg pro-recovery survival and function while reducing off-target effects (152).

## Open questions

A number of questions with translational relevance remain open in the field despite extensive investigation into the mechanisms of Treg function in the context of acute respiratory infection. Here, we highlight some of these questions and speculate on how to address them going forward.

### How does this rare immune cell subset comprising only 5% to 20% of lung CD4^+^ T cells exert such a profound effect on lung protection and regeneration following injury?

We hypothesize that temporally coordinated and spatially restricted Treg-specific inductive signals to key alveolar progenitor populations enable Tregs to exert a major impact on restoration of barrier function despite their rarity. Using single-cell RNA sequencing and lineage-tracing technologies, investigators have started to uncover the distinctive heterogeneity and cell-specific contribution of alveolar epithelial, mesenchymal, and endothelial cell subpopulations in lung regeneration (96, 98, 101, 102, 153–158). Future research can leverage systems biology approaches and spatial profiling technologies to investigate spatially coordinated gene expression between Tregs and KRT8^+^ transitional-state alveolar epithelial cells (also called pre-alveolar type 1 transitional cell state, alveolar differentiation intermediate, damage-associated transient progenitors, and intermediate alveolar epithelial cells), CAR4^+^ ECs, and *Col14a1*-expressing mesenchymal cells, potentially elucidating novel intercellular signaling and molecular pathways that drive lung repair. Mechanistically, the temporal aspect of different Treg functions can be investigated with a tamoxifen-inducible Cre recombinase system expressed from the *Foxp3* locus that is already in wide use (159). This system also allows tracing of *Foxp3^+^* cells to track lineage stability and resolve the spatial localization of Tregs (39).

### What are the cues that promote homing of Tregs to the lung and elicit their pro-recovery functions?

Extracellular matrix–driven (ECM-driven) mechanical signals influence immune cell migration and recruitment, including T cells (160), but how the local distribution of different ECM cellular components supports Treg accumulation and modulates Treg function during the inflammatory and fibroproliferative phases of lung injury remains unknown. Outside of CCR4- and CXCR3-mediated trafficking discussed above (127, 128), Treg-intrinsic mechanisms that promote homing to the injured lung are largely undefined. Whether and how the Treg TCR controls the Treg response to lung infection and injury also persists as an unresolved issue. Adoptive transfer of polyclonal splenic Tregs is sufficient to promote recovery from ongoing lung injury in lymphocyte-deficient (*Rag1^–/–^*) and Treg-deficient (diphtheria toxin–treated *Foxp3^DTR^*) mice (18–20, 25). It remains unclear whether a specific clone emerges after the transferred cells traffic to the lung. In a pivotal study by Arpaia and colleagues, induced ablation of the TCR on Tregs did not hinder their ability to produce tissue-protective AREG (23). In contrast, Tregs require TCR specificity to promote tissue regeneration following acute injury in muscle and visceral adipose tissue (161, 162). Detailed assessment of the TCR clonotypes that emerge in experimental and human respiratory infection will improve our understanding of the role of antigen-specific Tregs in the immune response to respiratory infection.

### Do subsets of Tregs with distinct pro-resolution and pro-repair functions exist, or do they emerge in specific contexts?

While bulk adoptive transfer of splenic Tregs is sufficient to promote recovery from experimental acute lung injury, there may be preexisting subsets of Tregs with specialized pro-recovery functions or dynamic processes that result in the emergence of a pro-recovery Treg subset from an otherwise homogenous population (163, 164). Which of these possibilities drives recovery from lung infection–induced injury remains unclear. As discussed above, IL-18 and IL-33 appear to signal through their receptors to induce the generation of reparative molecules, including AREG and possibly other growth factors and mediators. Accordingly, the IL-18 receptor may mark a reparative Treg subset (23, 165). Similarly, the IL-33 receptor subunit ST2 may serve as a marker of a reparative Treg subset in mice (23, 28, 29, 163, 164, 166). Important differences in ST2 and AREG expression between mouse and human Tregs may complicate identification of a reparative Treg subset and translation of ST2^+^ or AREG-expressing Tregs for therapeutic tissue protection and repair in patients (167). Moreover, the effects of IL-33 on Tregs are pleiotropic, enhancing their immunosuppressive function in the tumor and injured lung microenvironments (168–171). Finally, while AREG and KGF have garnered substantial attention as discussed above, other EGFR ligands and soluble growth factors may also promote lung repair. Treg subsets that preferentially generate these molecules — or spatially localize to lung niches that optimize the short-range function of Treg-derived soluble growth factors — remain to be fully defined. Going forward, single-cell profiling of the FOXP3^+^ cell population of the injured lung paired with spatial transcriptomics and proteomics will uncover heterogeneity and identify rare yet potentially potent cell subsets involved in distinct pro-recovery functions.

### How does the ontogeny of Tregs influence their functional characteristics?

While some markers (e.g., HELIOS and NRP1) may differentiate tTregs from pTregs in some settings, no known set of markers separates these cells by their developmental origin in all contexts (172–175). Functionally, pTregs and tTregs appear to occupy distinct immunological niches, with pTregs serving to maintain homeostasis at mucosal surfaces where they may regulate the microbiome to modulate the host response to influenza infection (176–181). Differentiating whether the Tregs that appear in the lung following injury derive from pTreg or tTreg origins is experimentally challenging. Mice with deletion of the *Foxp3* locus CNS1 do not generate pTregs and could serve to identify the contribution of pTregs versus tTregs in experimental lung injury (182). Finally, pTregs and tTregs carry distinct DNA methylation patterns at CNS0 and CNS2 (35, 183), which could be used to understand the contribution of each subset responding to lung injury. The role of epigenetic modifiers in these subsets to differentially modulate their function in the context of lung injury remains unknown (184).

### How can Tregs be optimized as a clinical immunotherapeutic strategy?

As detailed above, therapeutic translation of Tregs for use in promoting resolution of inflammation and repair of tissue damage following lung infection remains in its infant stages. Generating sufficient numbers of Tregs on a time scale that is aligned to clinical reality remains a challenge; off-the-shelf preparations can be used (27, 123), and strategies such as treatment with rapamycin have shown promise in promoting Treg purity in culture (125, 126). The potential for loss of FOXP3^+^ cell identity and conversion to a proinflammatory phenotype introduces additional difficulty to the standardizing of individual doses and the limiting of immune-mediated toxicity. In the inflamed lung, Tregs must rewire their metabolism to adapt to the hypoxic and nutrient-depleted microenvironment of the injured alveolar space (185). We demonstrated that mitochondria-generated metabolites shape Treg function, potentially by altering their DNA methylation profile (186). Hence, strategies to stabilize Treg identity in the face of metabolic stress, such as treatment with modulators of DNA methylation, could enhance the efficacy and safety of Treg-based cellular therapy (19, 134–136). CAR Treg technology could also be used to further optimize infused Tregs by improving their specificity and trafficking to tissues of interest (187). Advances in preparation of Treg therapies — such as the optimal number of infused cells, requirement for antigen recognition, and appropriate timing of the infusion — will open avenues to address specific disease states.

## Conclusions

Tregs exert diverse roles in regulating the immune response to respiratory infections, suppressing injurious inflammation during the primary antimicrobial response, and promoting repair and return to homeostatic conditions following the infection. To achieve the goal of restoring optimal lung function, Tregs affect multiple immune and nonimmune cell types, including neutrophils, macrophages, eosinophils, CD8^+^ and CD4^+^ T cells, and epithelial, endothelial, and mesenchymal cells. Dysregulation of this complex ensemble conducted by Tregs leads to increased inflammation, poor repair, and, ultimately, failed recovery from respiratory infections. Augmenting Treg responses is a potential therapeutic strategy for the treatment of severe acute respiratory infections that lead to substantial morbidity and mortality.

The relevance of Tregs in regulating the immune response to respiratory infections has been established mostly in mice. Yet there are important differences in the lung compartment anatomy and cellular heterogeneity between mice and humans (188, 189) that could hinder the interpretability and influence of translation-based discoveries in mice for human health. Future work will need to further delineate the mechanisms that contribute to Treg function in humans. Robust transcriptomic analysis of Tregs recruited to the lungs will help identify subsets more adept at specific roles currently attributed to Tregs in general. Subsequently, identifying signals that bias the Treg population toward these subsets will shed light on how to manipulate and optimize the Treg response to respiratory pathogens. Additional insights into the stabilization of Treg identity will further unlock their potential as a cellular therapy.

## Author contributions

The order of the 2 co–first authors was determined on the basis of their contributions to the writing of the manuscript.

## Figures and Tables

**Table 1:**
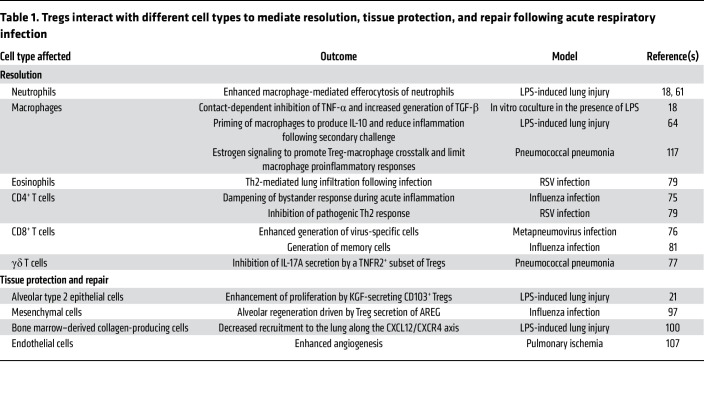
Tregs interact with different cell types to mediate resolution, tissue protection, and repair following acute respiratory infection

## Version 1 07/17/2023

Electronic publication

## References

[1] Torres A (2021). Pneumonia. Nat Rev Dis Primers.

[2] Gao CA (2022). Gearing up for battle: harnessing adaptive T cell immunity against gram-negative pneumonia. Front Cell Infect Microbiol.

[3] El Bcheraoui C (2018). Trends and patterns of differences in infectious disease mortality among US counties, 1980-2014. JAMA.

[4] Jin X (2021). Global burden of upper respiratory infections in 204 countries and territories, from 1990 to 2019. EClinicalMedicine.

[5] Cilloniz C (2022). Respiratory viruses: their importance and lessons learned from COVID-19. Eur Respir Rev.

[6] Cilloniz C (2019). Pure viral sepsis secondary to community-acquired pneumonia in adults: risk and prognostic factors. J Infect Dis.

[7] Jain S (2015). Community-acquired pneumonia requiring hospitalization among U.S. Adults. N Engl J Med.

[8] Khan YA (2021). Precision medicine and heterogeneity of treatment effect in therapies for ARDS. Chest.

[9] Matthay MA (2012). The acute respiratory distress syndrome. J Clin Invest.

[10] Zheng S (2020). Viral load dynamics and disease severity in patients infected with SARS-CoV-2 in Zhejiang province, China, January-March 2020: retrospective cohort study. BMJ.

[11] Yang L (2021). The signal pathways and treatment of cytokine storm in COVID-19. Signal Transduct Target Ther.

[12] Walsh KB (2011). Suppression of cytokine storm with a sphingosine analog provides protection against pathogenic influenza virus. Proc Natl Acad Sci U S A.

[13] Lee N (2011). Viral clearance and inflammatory response patterns in adults hospitalized for pandemic 2009 influenza A(H1N1) virus pneumonia. Antivir Ther.

[14] Endeman H (2011). Systemic cytokine response in patients with community-acquired pneumonia. Eur Respir J.

[15] Weinberg SE, Singer BD (2021). Toward a paradigm to distinguish distinct functions of FOXP3^+^ regulatory T cells. Immunohorizons.

[16] Maizels RM, Smith KA (2011). Regulatory T cells in infection. Adv Immunol.

[17] Nosbaum A (2016). Cutting edge: Regulatory T cells facilitate cutaneous wound healing. J Immunol.

[18] D’Alessio FR (2009). CD4+CD25+Foxp3+ Tregs resolve experimental lung injury in mice and are present in humans with acute lung injury. J Clin Invest.

[19] Singer BD (2015). Regulatory T cell DNA methyltransferase inhibition accelerates resolution of lung inflammation. Am J Respir Cell Mol Biol.

[20] Mock JR (2014). Foxp3+ regulatory T cells promote lung epithelial proliferation. Mucosal Immunol.

[21] Dial CF (2017). Foxp3^+^ regulatory T cell expression of keratinocyte growth factor enhances lung epithelial proliferation. Am J Respir Cell Mol Biol.

[22] Mock JR (2019). Transcriptional analysis of Foxp3+ Tregs and functions of two identified molecules during resolution of ALI. JCI Insight.

[23] Arpaia N (2015). A distinct function of regulatory T cells in tissue protection. Cell.

[24] Mock JR (2020). Impact of regulatory T cells on type 2 alveolar epithelial cell transcriptomes during resolution of acute lung injury and contributions of IFN-γ. Am J Respir Cell Mol Biol.

[25] Morales-Nebreda L (2021). Aging imparts cell-autonomous dysfunction to regulatory T cells during recovery from influenza pneumonia. JCI Insight.

[26] McKinley L (2006). Regulatory T cells dampen pulmonary inflammation and lung injury in an animal model of pneumocystis pneumonia. J Immunol.

[27] Gladstone DE (2020). Regulatory T cells for treating patients with COVID-19 and acute respiratory distress syndrome: two case reports. Ann Intern Med.

[28] Burzyn D (2013). A special population of regulatory T cells potentiates muscle repair. Cell.

[29] Kuswanto W (2016). Poor repair of skeletal muscle in aging mice reflects a defect in local, interleukin-33-dependent accumulation of regulatory T cells. Immunity.

[30] Tamosiuniene R (2011). Regulatory T cells limit vascular endothelial injury and prevent pulmonary hypertension. Circ Res.

[31] Walter JM (2018). Multidimensional assessment of alveolar T cells in critically ill patients. JCI Insight.

[32] Grant RA (2021). Circuits between infected macrophages and T cells in SARS-CoV-2 pneumonia. Nature.

[33] Joudi AM (2022). Epigenetic control of regulatory T cell stability and function: implications for translation. Front Immunol.

[34] Hsieh CS (2012). Selection of regulatory T cells in the thymus. Nat Rev Immunol.

[35] Kitagawa Y (2017). Guidance of regulatory T cell development by Satb1-dependent super-enhancer establishment. Nat Immunol.

[36] Ohkura N (2013). Development and maintenance of regulatory T cells. Immunity.

[37] Vahl JC (2014). Continuous T cell receptor signals maintain a functional regulatory T cell pool. Immunity.

[38] Levine AG (2014). Continuous requirement for the TCR in regulatory T cell function. Nat Immunol.

[39] Helmin KA (2020). Maintenance DNA methylation is essential for regulatory T cell development and stability of suppressive function. J Clin Invest.

[40] Brunkow ME (2001). Disruption of a new forkhead/winged-helix protein, scurfin, results in the fatal lymphoproliferative disorder of the scurfy mouse. Nat Genet.

[41] Hadaschik EN (2015). Regulatory T cell-deficient scurfy mice develop systemic autoimmune features resembling lupus-like disease. Arthritis Res Ther.

[42] Bennett CL (2001). The immune dysregulation, polyendocrinopathy, enteropathy, X-linked syndrome (IPEX) is caused by mutations of FOXP3. Nat Genet.

[43] Park JH (2020). Immune dysregulation, polyendocrinopathy, enteropathy, X-linked (IPEX) syndrome: a systematic review. Autoimmun Rev.

[44] Dominguez-Villar M, Hafler DA (2018). Regulatory T cells in autoimmune disease. Nat Immunol.

[45] Atif M (2020). Regulatory T cells in solid organ transplantation. Clin Transl Immunology.

[46] Liu H (2003). CD4+CD25+ regulatory T cells cure murine colitis: the role of IL-10, TGF-beta, and CTLA4. J Immunol.

[47] Li MO, Flavell RA (2008). Contextual regulation of inflammation: a duet by transforming growth factor-beta and interleukin-10. Immunity.

[48] Groux H (1997). A CD4+ T-cell subset inhibits antigen-specific T-cell responses and prevents colitis. Nature.

[49] Qureshi OS (2011). Trans-endocytosis of CD80 and CD86: a molecular basis for the cell-extrinsic function of CTLA-4. Science.

[50] Jain N (2010). Dual function of CTLA-4 in regulatory T cells and conventional T cells to prevent multiorgan autoimmunity. Proc Natl Acad Sci U S A.

[51] Ohta A, Sitkovsky M (2014). Extracellular adenosine-mediated modulation of regulatory T cells. Front Immunol.

[52] Deaglio S (2007). Adenosine generation catalyzed by CD39 and CD73 expressed on regulatory T cells mediates immune suppression. J Exp Med.

[53] Tanaka A, Sakaguchi S (2017). Regulatory T cells in cancer immunotherapy. Cell Res.

[54] Ohue Y, Nishikawa H (2019). Regulatory T (Treg) cells in cancer: can Treg cells be a new therapeutic target?. Cancer Sci.

[55] Abbas AK (2013). Regulatory T cells: recommendations to simplify the nomenclature. Nat Immunol.

[56] Toomer KH (2016). Developmental progression and interrelationship of central and effector regulatory T cell subsets. J Immunol.

[57] Miragaia RJ (2019). Single-cell transcriptomics of regulatory T cells reveals trajectories of tissue adaptation. Immunity.

[58] Siwicki M, Kubes P (2023). Neutrophils in host defense, healing, and hypersensitivity: dynamic cells within a dynamic host. J Allergy Clin Immunol.

[59] Walter JM (2019). Multidimensional assessment of the host response in mechanically ventilated patients with suspected pneumonia. Am J Respir Crit Care Med.

[60] Proto JD (2018). Regulatory T cells promote macrophage efferocytosis during inflammation resolution. Immunity.

[61] Pickens CO (2021). Bacterial superinfection pneumonia in patients mechanically ventilated for COVID-19 pneumonia. Am J Respir Crit Care Med.

[62] Guan T (2023). Regulatory T cell and macrophage crosstalk in acute lung injury: future perspectives. Cell Death Discov.

[63] Aggarwal NR (2014). Immunological priming requires regulatory T cells and IL-10-producing macrophages to accelerate resolution from severe lung inflammation. J Immunol.

[64] Fang S (2022). The role of interleukin-22 in lung health and its therapeutic potential for COVID-19. Front Immunol.

[65] de Araujo EF (2020). Pulmonary paracoccidioidomycosis in AhR deficient hosts is severe and associated with defective Treg and th22 responses. Sci Rep.

[66] Lin S (2014). Treg cells: a potential regulator for IL-22 expression?. Int J Clin Exp Pathol.

[67] Wang L (2012). BLT1-dependent alveolar recruitment of CD4(+)CD25(+) Foxp3(+) regulatory T cells is important for resolution of acute lung injury. Am J Respir Crit Care Med.

[68] Budinger GRS (2021). Distinctive features of severe SARS-CoV-2 pneumonia. J Clin Invest.

[69] Bruen C (2022). Auxora vs. placebo for the treatment of patients with severe COVID-19 pneumonia: a randomized-controlled clinical trial. Crit Care.

[70] Yero A (2021). Dynamics and epigenetic signature of regulatory T-cells following antiretroviral therapy initiation in acute HIV infection. EBioMedicine.

[71] Fulton RB (2010). Foxp3+ CD4 regulatory T cells limit pulmonary immunopathology by modulating the CD8 T cell response during respiratory syncytial virus infection. J Immunol.

[72] Betts RJ (2012). Influenza A virus infection results in a robust, antigen-responsive, and widely disseminated Foxp3+ regulatory T cell response. J Virol.

[73] Anghelina D (2009). Role of regulatory T cells in coronavirus-induced acute encephalitis. Virology.

[74] Walker MR (2005). De novo generation of antigen-specific CD4+CD25+ regulatory T cells from human CD4+CD25- cells. Proc Natl Acad Sci U S A.

[75] Rogers MC (2018). CD4^+^ regulatory T cells exert differential functions during early and late stages of the immune response to respiratory viruses. J Immunol.

[76] Xu R (2023). TNFR2^+^ regulatory T cells protect against bacteremic pneumococcal pneumonia by suppressing IL-17A-producing γδ T cells in the lung. Cell Rep.

[77] Raiden S (2014). Depletion of circulating regulatory T cells during severe respiratory syncytial virus infection in young children. Am J Respir Crit Care Med.

[78] Durant LR (2013). Regulatory T cells prevent Th2 immune responses and pulmonary eosinophilia during respiratory syncytial virus infection in mice. J Virol.

[79] Lin PH (2018). Vaccine-induced antigen-specific regulatory T cells attenuate the antiviral immunity against acute influenza virus infection. Mucosal Immunol.

[80] Brincks EL (2013). Antigen-specific memory regulatory CD4^+^Foxp3^+^ T cells control memory responses to influenza virus infection. J Immunol.

[81] Ferreira C (2020). Type 1 T_reg_ cells promote the generation of CD8^+^ tissue-resident memory T cells. Nat Immunol.

[82] Brunstein CG (2011). Infusion of ex vivo expanded T regulatory cells in adults transplanted with umbilical cord blood: safety profile and detection kinetics. Blood.

[83] Di Ianni M (2011). Tregs prevent GVHD and promote immune reconstitution in HLA-haploidentical transplantation. Blood.

[84] Lopez-Otin C, Kroemer G (2021). Hallmarks of health. Cell.

[85] Basil MC (2020). The cellular and physiological basis for lung repair and regeneration: past, present, and future. Cell Stem Cell.

[86] Kotton DN, Morrisey EE (2014). Lung regeneration: mechanisms, applications and emerging stem cell populations. Nat Med.

[87] Konkimalla A (2022). Lung regeneration: cells, models, and mechanisms. Cold Spring Harb Perspect Biol.

[88] Mock JR (2020). Effects of IFN-γ on immune cell kinetics during the resolution of acute lung injury. Physiol Rep.

[89] Kim JM (2007). Regulatory T cells prevent catastrophic autoimmunity throughout the lifespan of mice. Nat Immunol.

[90] Palmiter RD (1987). Cell lineage ablation in transgenic mice by cell-specific expression of a toxin gene. Cell.

[91] McAuley DF (2017). Keratinocyte growth factor for the treatment of the acute respiratory distress syndrome (KARE): a randomised, double-blind, placebo-controlled phase 2 trial. Lancet Respir Med.

[92] Monticelli LA (2011). Innate lymphoid cells promote lung-tissue homeostasis after infection with influenza virus. Nat Immunol.

[93] Monticelli LA (2015). IL-33 promotes an innate immune pathway of intestinal tissue protection dependent on amphiregulin-EGFR interactions. Proc Natl Acad Sci U S A.

[94] Krishnan S (2018). Amphiregulin-producing γδ T cells are vital for safeguarding oral barrier immune homeostasis. Proc Natl Acad Sci U S A.

[95] Ito M (2019). Brain regulatory T cells suppress astrogliosis and potentiate neurological recovery. Nature.

[96] Kaiser KA (2023). Regulation of the alveolar regenerative niche by amphiregulin-producing regulatory T cells. J Exp Med.

[97] Harb H (2021). Notch4 signaling limits regulatory T-cell-mediated tissue repair and promotes severe lung inflammation in viral infections. Immunity.

[98] Zepp JA (2017). Distinct mesenchymal lineages and niches promote epithelial self-renewal and myofibrogenesis in the lung. Cell.

[99] Garibaldi BT (2013). Regulatory T cells reduce acute lung injury fibroproliferation by decreasing fibrocyte recruitment. Am J Respir Cell Mol Biol.

[100] Ding BS (2011). Endothelial-derived angiocrine signals induce and sustain regenerative lung alveolarization. Cell.

[101] Niethamer TK (2020). Defining the role of pulmonary endothelial cell heterogeneity in the response to acute lung injury. Elife.

[102] Vila Ellis L (2020). Epithelial Vegfa specifies a distinct endothelial population in the mouse lung. Dev Cell.

[103] Luznik Z (2020). Regulatory T cells in angiogenesis. J Immunol.

[104] Facciabene A (2011). Tumour hypoxia promotes tolerance and angiogenesis via CCL28 and T(reg) cells. Nature.

[105] Leung OM (2018). Regulatory T cells promote apelin-mediated sprouting angiogenesis in type 2 diabetes. Cell Rep.

[106] D’Alessio FR (2015). Lung angiogenesis requires CD4(+) forkhead homeobox protein-3(+) regulatory T cells. Am J Respir Cell Mol Biol.

[107] Iuliano AD (2018). Estimates of global seasonal influenza-associated respiratory mortality: a modelling study. Lancet.

[108] Torres Acosta MA, Singer BD (2020). Pathogenesis of COVID-19-induced ARDS: implications for an ageing population. Eur Respir J.

[109] Goronzy JJ, Weyand CM (2017). Successful and maladaptive T cell aging. Immunity.

[110] Sun X (2022). Longitudinal analysis reveals age-related changes in the T cell receptor repertoire of human T cell subsets. J Clin Invest.

[111] Bapat SP (2015). Depletion of fat-resident Treg cells prevents age-associated insulin resistance. Nature.

[113] Offner PJ (1999). Male gender is a risk factor for major infections after surgery. Arch Surg.

[114] Loeb M (1999). Risk factors for pneumonia and other lower respiratory tract infections in elderly residents of long-term care facilities. Arch Intern Med.

[115] Dadhwal K (2022). Severe COVID-19 pneumonia in an intensive care setting and comparisons with historic severe viral pneumonia due to other viruses. Clin Respir J.

[116] Xiong Y (2020). Estradiol resolves pneumonia via ERβ in regulatory T cells. JCI Insight.

[117] Bluestone JA (2015). Type 1 diabetes immunotherapy using polyclonal regulatory T cells. Sci Transl Med.

[118] Trzonkowski P (2009). First-in-man clinical results of the treatment of patients with graft versus host disease with human ex vivo expanded CD4+CD25+CD127- T regulatory cells. Clin Immunol.

[119] Mathew JM (2018). A phase I clinical trial with ex vivo expanded recipient regulatory t cells in living donor kidney transplants. Sci Rep.

[120] Sawitzki B (2020). Regulatory cell therapy in kidney transplantation (the ONE Study): a harmonised design and analysis of seven non-randomised, single-arm, phase 1/2A trials. Lancet.

[121] Harden PN (2021). Feasibility, long-term safety, and immune monitoring of regulatory T cell therapy in living donor kidney transplant recipients. Am J Transplant.

[122] Brunstein CG (2016). Umbilical cord blood-derived T regulatory cells to prevent GVHD: kinetics, toxicity profile, and clinical effect. Blood.

[123] Gladstone DE Randomized, double blinded, placebo controlled trial of allogeneic cord blood T-regulatory cell for treatment of COVID-19 ARDS. Blood Adv.

[124] Morales-Nebreda L (2020). CoRESTed development of regulatory T cells. J Clin Invest.

[125] Strauss L (2007). Selective survival of naturally occurring human CD4+CD25+Foxp3+ regulatory T cells cultured with rapamycin. J Immunol.

[126] Fraser H (2018). A rapamycin-based GMP-compatible process for the isolation and expansion of regulatory T cells for clinical trials. Mol Ther Methods Clin Dev.

[127] Sather BD (2007). Altering the distribution of Foxp3(+) regulatory T cells results in tissue-specific inflammatory disease. J Exp Med.

[128] Koch MA (2009). The transcription factor T-bet controls regulatory T cell homeostasis and function during type 1 inflammation. Nat Immunol.

[129] Rana J (2021). CAR- and TRuC-redirected regulatory T cells differ in capacity to control adaptive immunity to FVIII. Mol Ther.

[130] Imura Y (2020). CD19-targeted CAR regulatory T cells suppress B cell pathology without GvHD. JCI Insight.

[131] Boardman DA (2023). Flagellin-specific human CAR Tregs for immune regulation in IBD. J Autoimmun.

[132] Zhou X (2009). Instability of the transcription factor Foxp3 leads to the generation of pathogenic memory T cells in vivo. Nat Immunol.

[133] McGrath-Morrow SA (2018). DNA methylation regulates the neonatal CD4^+^ T-cell response to pneumonia in mice. J Biol Chem.

[134] Lu CH (2016). DNA methyltransferase inhibitor promotes human CD4(+)CD25(h)FOXP3(+) regulatory T lymphocyte induction under suboptimal TCR stimulation. Front Immunol.

[135] Chan MW (2014). Low-dose 5-aza-2′-deoxycytidine pretreatment inhibits experimental autoimmune encephalomyelitis by induction of regulatory T cells. Mol Med.

[136] Han P (2021). Low-dose decitabine modulates T-cell homeostasis and restores immune tolerance in immune thrombocytopenia. Blood.

[137] Lal G (2009). Epigenetic regulation of Foxp3 expression in regulatory T cells by DNA methylation. J Immunol.

[138] Samanta A (2008). TGF-beta and IL-6 signals modulate chromatin binding and promoter occupancy by acetylated FOXP3. Proc Natl Acad Sci U S A.

[139] Yue X (2016). Control of Foxp3 stability through modulation of TET activity. J Exp Med.

[140] Sasidharan Nair V (2016). Vitamin C facilitates demethylation of the Foxp3 enhancer in a Tet-dependent manner. J Immunol.

[141] Rothhammer V, Quintana FJ (2019). The aryl hydrocarbon receptor: an environmental sensor integrating immune responses in health and disease. Nat Rev Immunol.

[142] Abdulla OA (2021). The ability of AhR ligands to attenuate delayed type hypersensitivity reaction is associated with alterations in the gut microbiota. Front Immunol.

[143] Allen BL, Taatjes DJ (2015). The Mediator complex: a central integrator of transcription. Nat Rev Mol Cell Biol.

[144] Tsutsui T (2013). Mediator complex recruits epigenetic regulators via its two cyclin-dependent kinase subunits to repress transcription of immune response genes. J Biol Chem.

[145] Akamatsu M (2019). Conversion of antigen-specific effector/memory T cells into Foxp3-expressing T_reg_ cells by inhibition of CDK8/19. Sci Immunol.

[146] Cheng G (2013). IL-2R signaling is essential for functional maturation of regulatory T cells during thymic development. J Immunol.

[147] Malek TR, Bayer AL (2004). Tolerance, not immunity, crucially depends on IL-2. Nat Rev Immunol.

[148] Koreth J (2011). Interleukin-2 and regulatory T cells in graft-versus-host disease. N Engl J Med.

[149] Krieg C (2010). Improved IL-2 immunotherapy by selective stimulation of IL-2 receptors on lymphocytes and endothelial cells. Proc Natl Acad Sci U S A.

[150] Hirai T (2021). Selective expansion of regulatory T cells using an orthogonal IL-2/IL-2 receptor system facilitates transplantation tolerance. J Clin Invest.

[151] Zhang Q (2021). A human orthogonal IL-2 and IL-2Rβ system enhances CAR T cell expansion and antitumor activity in a murine model of leukemia. Sci Transl Med.

[152] Hernandez R (2022). Engineering IL-2 for immunotherapy of autoimmunity and cancer. Nat Rev Immunol.

[153] Zacharias WJ (2018). Regeneration of the lung alveolus by an evolutionarily conserved epithelial progenitor. Nature.

[154] Strunz M (2020). Alveolar regeneration through a Krt8^+^ transitional stem cell state that persists in human lung fibrosis. Nat Commun.

[155] Nabhan AN (2018). Single-cell Wnt signaling niches maintain stemness of alveolar type 2 cells. Science.

[156] Kobayashi Y (2020). Persistence of a regeneration-associated, transitional alveolar epithelial cell state in pulmonary fibrosis. Nat Cell Biol.

[157] Choi J (2020). Inflammatory signals induce AT2 cell-derived damage-associated transient progenitors that mediate alveolar regeneration. Cell Stem Cell.

[158] Reyfman PA (2019). Single-cell transcriptomic analysis of human lung provides insights into the pathobiology of pulmonary fibrosis. Am J Respir Crit Care Med.

[159] Rubtsov YP (2010). Stability of the regulatory T cell lineage in vivo. Science.

[160] Salmon H (2012). Matrix architecture defines the preferential localization and migration of T cells into the stroma of human lung tumors. J Clin Invest.

[161] Fernandes RA (2020). Discovery of surrogate agonists for visceral fat Treg cells that modulate metabolic indices in vivo. Elife.

[162] Cho J (2019). T cell receptor specificity drives accumulation of a reparative population of regulatory T cells within acutely injured skeletal muscle. Proc Natl Acad Sci U S A.

[163] Delacher M (2021). Single-cell chromatin accessibility landscape identifies tissue repair program in human regulatory T cells. Immunity.

[164] Delacher M (2017). Genome-wide DNA-methylation landscape defines specialization of regulatory T cells in tissues. Nat Immunol.

[165] Varanasi SK (2018). Role of IL-18 induced Amphiregulin expression on virus induced ocular lesions. Mucosal Immunol.

[166] Sakai R (2021). Kidney GATA3^+^ regulatory T cells play roles in the convalescence stage after antibody-mediated renal injury. Cell Mol Immunol.

[167] Lam AJ (2019). Innate control of tissue-reparative human regulatory T cells. J Immunol.

[168] Nascimento DC (2017). IL-33 contributes to sepsis-induced long-term immunosuppression by expanding the regulatory T cell population. Nat Commun.

[169] Liu Q (2019). IL-33-mediated IL-13 secretion by ST2^+^ Tregs controls inflammation after lung injury. JCI Insight.

[170] Siede J (2016). IL-33 receptor-expressing regulatory T cells are highly activated, Th2 biased and suppress CD4 T cell proliferation through IL-10 and TGFβ release. PLoS One.

[171] Pastille E (2019). The IL-33/ST2 pathway shapes the regulatory T cell phenotype to promote intestinal cancer. Mucosal Immunol.

[172] Savage PA (2020). Regulatory T cell development. Annu Rev Immunol.

[173] Singh K (2015). Concomitant analysis of Helios and neuropilin-1 as a marker to detect thymic derived regulatory T cells in naïve mice. Sci Rep.

[174] Elkord E (2016). Helios should not be cited as a marker of human thymus-derived Tregs. Commentary: Helios(+) and helios(-) cells coexist within the natural FOXP3(+) T regulatory cell subset in humans. Front Immunol.

[175] Szurek E (2015). Differences in expression level of helios and neuropilin-1 do not distinguish thymus-derived from extrathymically-induced CD4^+^Foxp3^+^ regulatory T cells. PLoS One.

[176] Oh JZ (2014). TLR5-mediated sensing of gut microbiota is necessary for antibody responses to seasonal influenza vaccination. Immunity.

[177] Campbell C (2018). Extrathymically generated regulatory T cells establish a niche for intestinal border-dwelling bacteria and affect physiologic metabolite balance. Immunity.

[178] Chinen T (2010). A critical role for regulatory T cell-mediated control of inflammation in the absence of commensal microbiota. J Exp Med.

[179] Josefowicz SZ (2012). Extrathymically generated regulatory T cells control mucosal TH2 inflammation. Nature.

[180] Rosshart SP (2017). Wild mouse gut microbiota promotes host fitness and improves disease resistance. Cell.

[181] Ichinohe T (2011). Microbiota regulates immune defense against respiratory tract influenza A virus infection. Proc Natl Acad Sci U S A.

[182] Zheng Y (2010). Role of conserved non-coding DNA elements in the Foxp3 gene in regulatory T-cell fate. Nature.

[183] Ohkura N (2012). T cell receptor stimulation-induced epigenetic changes and Foxp3 expression are independent and complementary events required for Treg cell development. Immunity.

[184] Ohkura N (2011). FOXP3+ regulatory T cells: control of FOXP3 expression by pharmacological agents. Trends Pharmacol Sci.

[185] Singer BD, Chandel NS (2019). Immunometabolism of pro-repair cells. J Clin Invest.

[186] Weinberg SE (2019). Mitochondrial complex III is essential for suppressive function of regulatory T cells. Nature.

[187] Arjomandnejad M (2022). CAR-T regulatory (CAR-Treg) cells: engineering and applications. Biomedicines.

[188] Kadur Lakshminarasimha Murthy P (2022). Human distal lung maps and lineage hierarchies reveal a bipotent progenitor. Nature.

[189] Basil MC, Morrisey EE (2020). Lung regeneration: a tale of mice and men. Semin Cell Dev Biol.

